# Bidirectional high-speed optical wireless communication with tunable large field of view assisted by liquid crystal metadevice

**DOI:** 10.1515/nanoph-2024-0434

**Published:** 2024-09-27

**Authors:** Mian Wu, Chao Yang, Yuhan Gong, Lin Wu, Ming Luo, Ying Qiu, Yongquan Zeng, Zile Li, Zichen Liu, Chao Li, Hanbing Li, Xi Xiao, Zhixue He, Guoxing Zheng, Shaohua Yu, Jin Tao

**Affiliations:** State Key Laboratory of Optical Communication Technologies and Networks, China Information Communication Technologies Group Corporation (CICT), Wuhan, China; Peng Cheng Laboratory, Shenzhen, China; Electronic Information School, Wuhan University, Wuhan, China; National Information Optoelectronics Innovation Center, Wuhan, China

**Keywords:** metasurfaces, optical wireless communication, beam steering, deflection magnification, liquid crystal

## Abstract

Beam-steered infrared (IR) light communication has gained tremendous attention as one of the solutions of congested wireless communication traffic. High performance active beam-steering devices play a crucial role in data allocation and exchange. Conventional beam-steering devices such as spatial light modulator (SLM) and micro-electrical mechanical system (MEMS) mirror and the current emerging nonmechanical beam-steering metasurface-based devices are challenging to realize a large tunable steering angle beyond several degrees, which significantly hinders the spatial application of optical wireless communications (OWC). Herein, an angle-magnified liquid crystal (LC) metadevice consisting of LC metasurfaces and a liquid crystal on silicon (LCoS) is proposed to realize active beam steering with a tunable large field of view (FOV). Based on the angle-magnified tunable LC metadevice, an intelligent bidirectional high-speed OWC system is experimentally demonstrated, achieving an actively enlarged FOV of 20° × 20°, with a data rate of 200 Gbps over the S/C/L band for both uplink and downlink transmission over a propagation distance of 1.5 m in free space. The proposed OWC system opens a new avenue for the future high performance wireless data transmission.

## 1 Introduction

In the past decade, information explosion caused by the dramatically growing number of mobile devices and the rapid development of the Internet of Things have been challenging the wireless communication traffic. As a promising solution, infrared (IR) optical wireless communications (OWC) have drawn significant attention for its ultra-high bandwidth of unregulated spectrum, electromagnetic interference immunity, low cost, and high data traffic [1], [2], [3], [4]. More importantly, it is independent of light illumination system of the architecture and compatible with the well-established optical fiber communication infrastructure in S + C + L band (1,460–1,625 nm). The high directivity of the propagating laser beam enables the connectivity among stationary or moving points with high information capacity and good data security. Consequently, the IR beam-steering technology plays a pivotal role in the energy-efficient and high-speed beam-steered IR light communication. Traditional beam steering by micro-electrical mechanical system (MEMS) mirror [5], [6], [7], spatial light modulator (SLM) [8], [9], diffraction gratings [10], [11], and arrayed waveguide gratings [12] cannot simultaneously realize beam steering with a large field of view (FOV), multi-subbeams, and tunability.

Metasurface is a kind of artificial two-dimensional material that is composed of periodic subwavelength metallic or dielectric unit structures, which has excellent capability to independently control amplitude, phase, polarization, spectrum, and momentum of waves [13], [14], [15], [16], [17], [18], [19], [20], [21], [22], [23], [24], [25], [26], [27]. Recently, the metasurface-based devices have shown strong wave control ability in the field of OWC. Transmissive dielectric metasurfaces were used to convert orthogonal polarization into high-order vectorial modes or transit fundamental spatial modes into higher-order spatial modes for space division multiplexed system at telecommunication band [28], [29]. A 20 Gbps 1D point-to-point OWC link with a FOV of 35° was experimentally enabled by a passively field-programmable reflective metasurface, which steered beams through polarization switching between normal and abnormal reflections [30]. A reflective beam splitting metasurface was demonstrated to support 2D point-to-multipoint full-duplex broadcasting communication system with the FOV angles up to ±40°, and data rates of 100 and 10 Gbps for the downstream and upstream, respectively [31]. A silicon metasurface-assisted bidirectional multichannel optical wireless system with coherent modulation and reception was experimentally demonstrated for 100 Gbps signal transmission over 2 m free space distance [32]. A full-color circular autofocusing airy beam metasurface is designed with a high polarization conversion efficiency, enabling an increased data rate and reliable 4 K video transmission in wavelength division multiplexing base on underwater wireless optical communication data link [33]. Recently, we presented an ultracompact metasurface assisted with an SLM for optical broadcasting communication system with nine broadcasting areas and a large FOV of 20° × 20°, where each beam can be dynamically steered in each area at a data rate of 10 Gbps [34]. Although the beam can be flexibly steered in each designed area, the steering angle is still limited up to 3° due to the micrometer scale size of the liquid crystal (LC) pixel. Larger steering angles were acquired using integrated metasurfaces, such as LC-integrated metasurfaces [23], [24], where metasurfaces were infiltered in LC with pixel pitch ∼1 μm through refractive index tuning or abrupt phase change, both enabled by voltage modulation [35], [36]. Two transmissive silicon cascaded metasurfaces were experimentally demonstrated for a continuous two-dimensional beam-tuning for 1,064 nm light by mechanical lateral translation of the metasurfaces [37]. A rotary doublet and triplet varifocal metadevices consisting of cascaded metasurfaces were demonstrated for full manipulating capacity of the THz beam’s propagation direction and coverage area [38]. However, these schemes need complex design, fabrication, or delicate alignment.

In this work, an LC metadevice consisting of LC metasurfaces and a liquid crystal on silicon (LCoS) is proposed, which realizes active beam steering with a tunable large FOV of 20° × 20°, linearly magnifying the 7° × 7° FOV of LCoS devices by 3 times and maintaining good collimation of steering beams. Based on the angle-magnified tunable LC metadevice, an intelligent bidirectional high-speed optical wireless communication is experimentally demonstrated, achieving a magnified FOV of 20° × 20°, with a data rate of 200 Gbps over the S/C/L band for both uplink and downlink transmission over a free space propagation distance of 1.5 m. The bit error rates (BERs) for bidirectional point-to-point link are below the soft decision forward error correction (FEC) limit of 2.4 × 10^−2^. The proposed LC metadevice–assisted intelligent high-speed OWC system suggests a practical approach for high-performance OWC, which is compatible with current optical architecture and commercial devices.

## 2 Results

### 2.1 Design of the optical characterization of the liquid crystal metadevice

**Figure 1:**
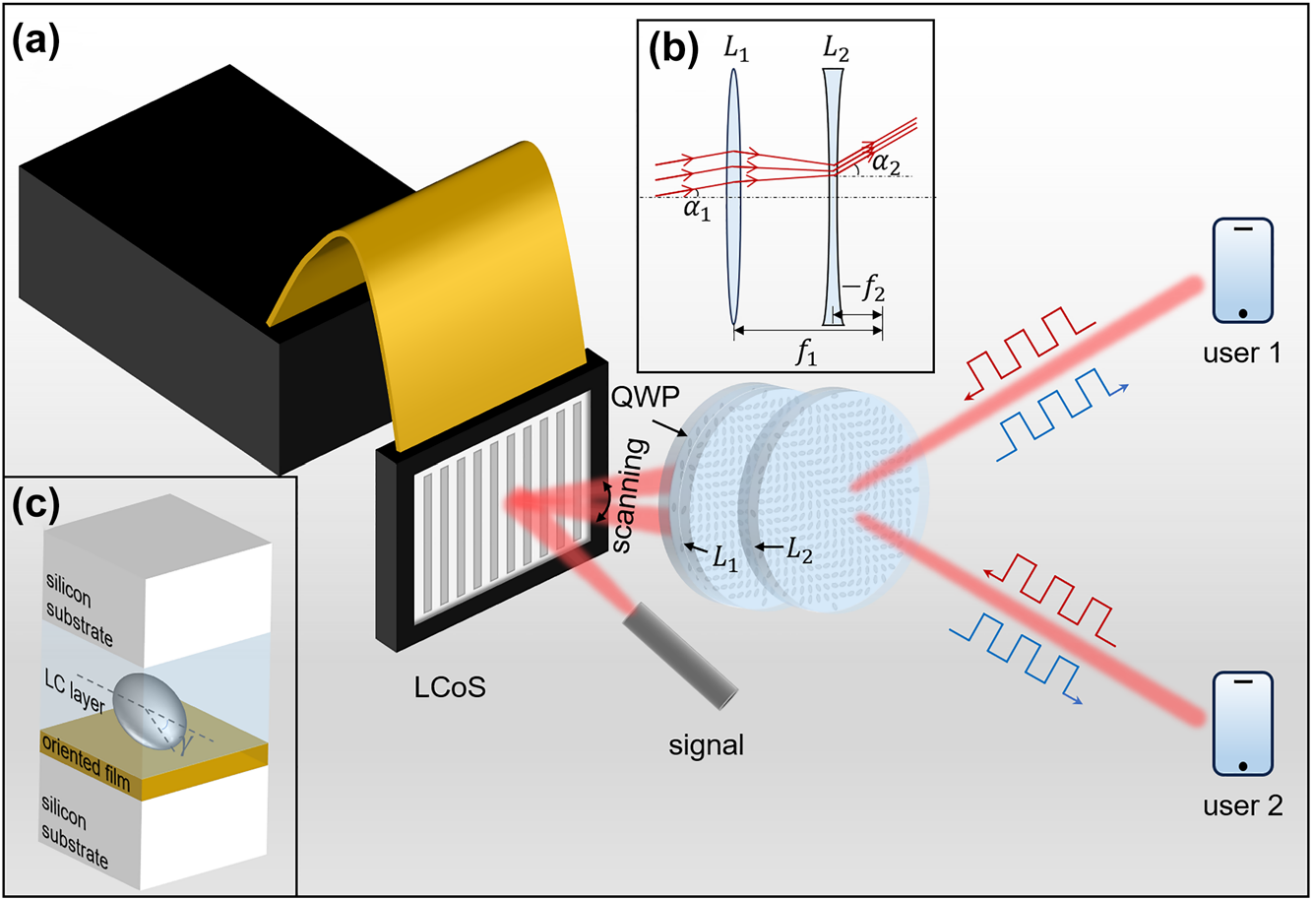
Schematic of the LC metadevice–assisted OWC system with tunable large FOV. (a) High-speed OWC system using LC metadevice with tunable and magnified FOV. (b) Principal diagram of the LC metalenses to enlarge the FOV. (C) Unit cell of the sandwich LC metasurface structure.

Figure 1(a) illustrates the schematic of bidirectional high-speed optical wireless communication system with tunable large FOV between the station and users assisted by LC metadevice. The proposed LC metadevice consists of an LCoS and a set of LC metasurfaces, which fully utilizes the tunability of LCoS and the controlled angular magnification of LC metasurface set simultaneously. The LC metasurface set includes an LC metalens *L*_1_ integrated with an LC quarter-wave plate (QWP) and another LC metalens *L*_2_, whose focal lengths are denoted as *f*_1_ and *f*_2_. As shown in Figure 1(b), the two metalenses work as a convex and a concave lens, spaced apart by the sum of focal lengths *f*_1_ + *f*_2_ to be confocal. In this way, the collimated incident beam will be outputted with a deflection angle magnified by *M* times, and *M* equals to −*f*_1_/*f*_2_. As a linear polarized signal beam is cast onto the metadevice, it is firstly modulated by LCoS, which is capable of 2D beam scanning within a small FOV. Later, the beam transmits through the LC meatasurface set, where the deflection beam is transferred into a left circular polarized (LCP) one through the LC QWP and is linearly and continuously magnified in the deflection angle and 2D scanning FOV through metalenses *L*_1_ and *L*_2_. Finally, the signal beam is sent to the dynamic moving users.

For better application, conventional lens counterparts with equivalent NA around 0.2 are not taken into consideration for its significantly curved surfaces, increased thickness, greater weight, and complexity of aberration correlation, while metalenses boasts flat surface, thin and compact factor, precise pointwise phase modulation, and high degree of design freedom, as well as capability to mitigate the aberration. The proposed LC metasurfaces are composed of the sandwich structured unit cells, which generally contain two silicon substrates, an oriented film, and the key functional LC layer as shown in Figure 1(c). The three LC metasurfaces are designed by different principles with precise and continuous phase regulation without increasing the processing complexity. The LC QWP is designed by accumulating the thickness of birefringent LC layer to obtain a propagation phase difference of π/2 or its odd multiples between ordinary light and extraordinary light. And the LC metalenses are designed via Pancharatnam–Berry phase modulation by varying the orientation angle *γ* of pixeled LC directors under LCP illumination to produce 2*γ* phase shifts, realizing a full 2π multilevel phase shift for beam steering. For polarization transform and angle magnification, the orientation angle distribution of LC directors of LC QWP is uniform, while that of the two metalenses are both characterized with quadratic profiles, whose corresponding phase distribution is as follows:

$$\varphi \left(r\right)=-\frac{2\pi }{\lambda }\left[\sqrt{r^{2}+f^{2}}-f\right]$$

where *r*, *λ*, and *f* are the distance to the metalens center, the design wavelength, and the focal length, respectively. It is worth mentioned that the LC metalenses function as expected only when incidence onto them is LCP light, so LC QWP is essential. Otherwise, if the incidence is right circular polarized (RCP), the phase shift will be inversed so that *L*_1_ and *L*_2_ will get opposite focal length, respectively, which breaks the confocal state of the two metalenses.

In physical realization, the LC QWP is integrated onto the front substrate of *L*_1_ to form a three-substrate structure, as shown in Figure 1(a). Given the limited deflection angle of LCoS, the effective apertures are set to be 6 mm for *L*_1_ and 4 mm for *L*_2_. The focal length *f*_1_ is designed as 21 mm and *f*_2_ is −7 mm at a working wavelength of 1,550 nm, so the magnification factor *M* is 3. To ensure at least 10 phase levels for the edge periods, the two lenses are designed with different pixel sizes, 0.91 × 0.91 μm^2^ for the front lens *L*_1_ and 0.47 × 0.47 μm^2^ for the back lens *L*_2_, so *L*_1_ has 6,593 pixels in diameter and *L*_2_ has 8,510 pixels in diameter.

**Figure 2:**
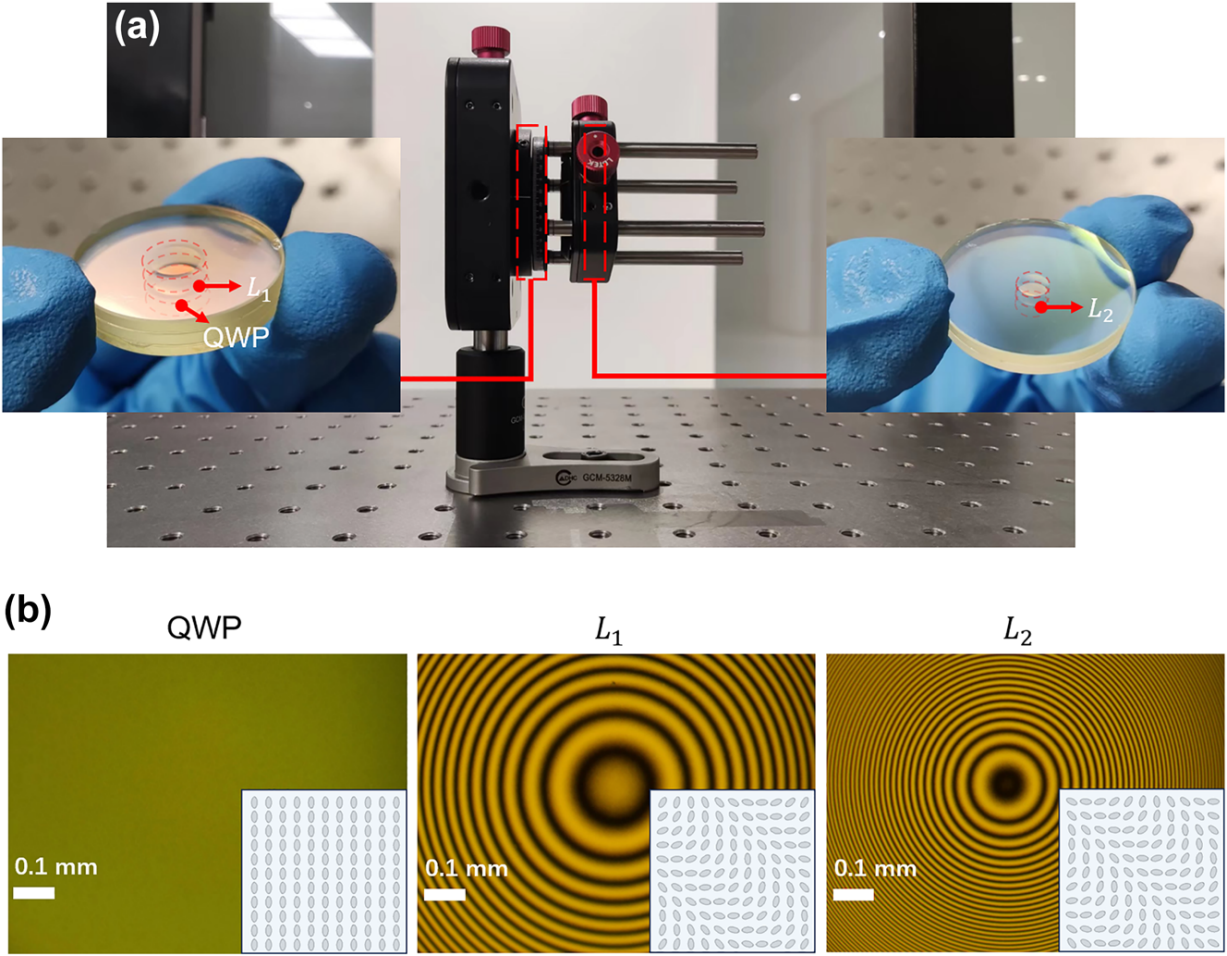
The fabricated LC metasurface set. (a) Physical diagram of the packaged LC metasurface set. The insets show the two fabricated bare samples. (b) Polarizing microscopic images of QWP, *L*_1_, and *L*_2_. The corresponding partial orientation distribution of LC directors is shown in the insets. Scale bar: 0.1 mm.

Figure 2(a) shows the photograph of the packaged LC metasurface set. The fabricated three LC metasurfaces are separated into two parts: a three-substrate structure containing LC QWP, and *L*_1_ shown in the left inset, and a two-substrate structure containing *L*_2_ depicted in the right inset. Figure 2(b) shows the optical polarizing microscopic images of LC QWP, *L*_1_, and *L*_2_, respectively. At the bottom-right corner of the insets in each image, partial orientation distributions of LC directors are displayed. As can be seen, the LC QWP has a uniform orientation distribution, while *L*_1_ and *L*_2_ have different but similar orientation distributions. It indicates the different design principles of them.

**Figure 3:**
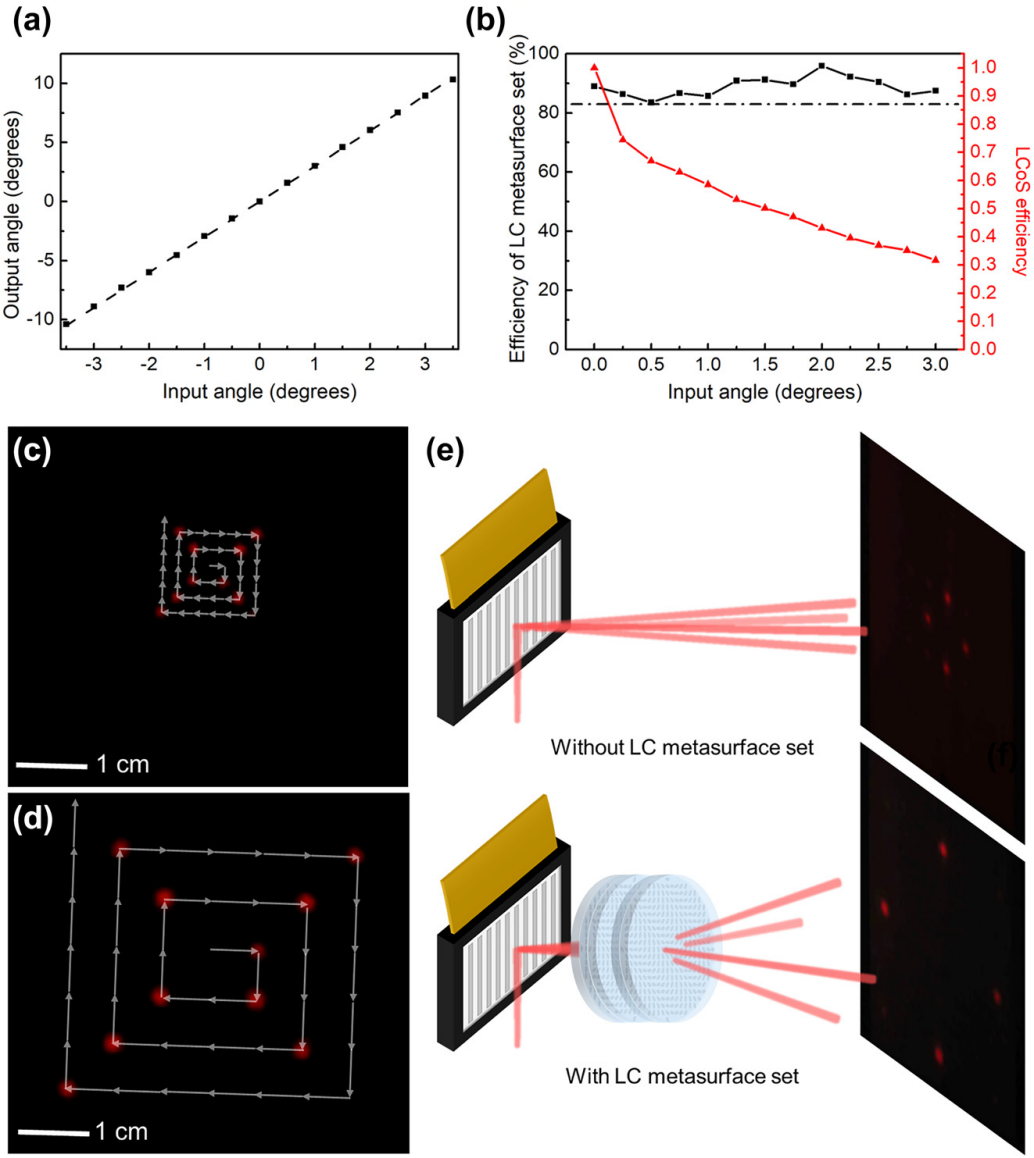
Characterization of the LC metadevice. (a) The measured results for the linear angle magnification ability. The slope of the fitting line (dotted line) is 3. (b) The transmission efficiency of LC metasurface set (black line) and the modulation efficiency of LCoS (red line) for different incident angles. The dotted line marks the efficiency of LC metasurface set of 83 %. (c) Tracks of a moving light spot modulated by LCoS only, on IR sensitive cards 0.25 m away. (d) Tracks of a moving light spot modulated and enlarged by LCoS and LC metasurface set, on IR sensitive cards 0.25 m away. (e) Point-to-multipoint beam steering displayed on IR sensitive cards 0.25 m away without LC metasurface set (upper) and with LC metasurface set (lower).

Next, we experimentally characterized the optical performance of the LC metadevice for tunable and large FOV. A laser beam at the operation wavelength of 1,550 nm through a collimator illuminated the reflective plane of LCoS with an incident angle of 45°, which enabled a configurable FOV of 7°, from −3.5° to 3.5°. The measured results for the linear angle magnification ability are shown in Figure 3(a), where the fitting line with a slope of 3 matches well with the experimental results. In other words, for the beam incident onto the LC metadevice rather than only incident onto the LCoS, the deflection angles at the output ports were magnified by three times, thus realizing a continuously adjustable FOV of 21°. The efficiencies were measured using an IR spatial optical detector as illustrated in Figure 3(b). For different incident angles, the LC metasurface set shows a stable and high transmission efficiency above 83 %. The overall efficiencies of LCoS are determined by the reflectivity and the modulation efficiency. At the incidence wavelength of 1,550 nm with LCoS-aligned polarization, the reflectivity of LCoS is 66.7 %, and the modulation efficiency decreases rapidly with increasing incident angles. It is because the initial reflection angle is larger than the working reflection angle of LCoS, which is a compromise of the excessive occlusion caused by the bulky package of the LC metasurface set. This can be eliminated by reducing the packaging footprint.

To evaluate the continuous 2D beam-steering function of the LC metadevice, we recorded the tracks of a deflected light spot, which is dynamically modulated by LCoS only or by the whole LC metadevice on IR sensitive cards 0.25 m away from the LCoS, as illustrated in Figure 3(c) and (d). The gray arrows indicate the moving paths of the light spots. Notably, it clearly indicates the good continuity and flexibility of 2D beam steering of LC metadevice, in addition to the linear magnification of 2D FOV. The complete videos of 2D beam-steering processes are provided in the Supporting Information. Apart from point-to-point beam steering, the proposed LC metadevice can also be utilized for point-to-multipoint beam steering. Figure 3(e) shows the four-beam steering displayed on IR sensitive cards 0.25 m away from LCoS, where the upper inset is multibeam steering tuned by LCoS only, while the lower inset is multibeam steering modulated by the whole LC metadevice. As can be seen, the expanded tunable FOV by LC metadevice brings more feasibilities for optical broadcasting system with large FOV.

### 2.2 Experiments of bidirectional high-speed optical wireless communication assisted by liquid crystal metadevice

**Figure 4:**
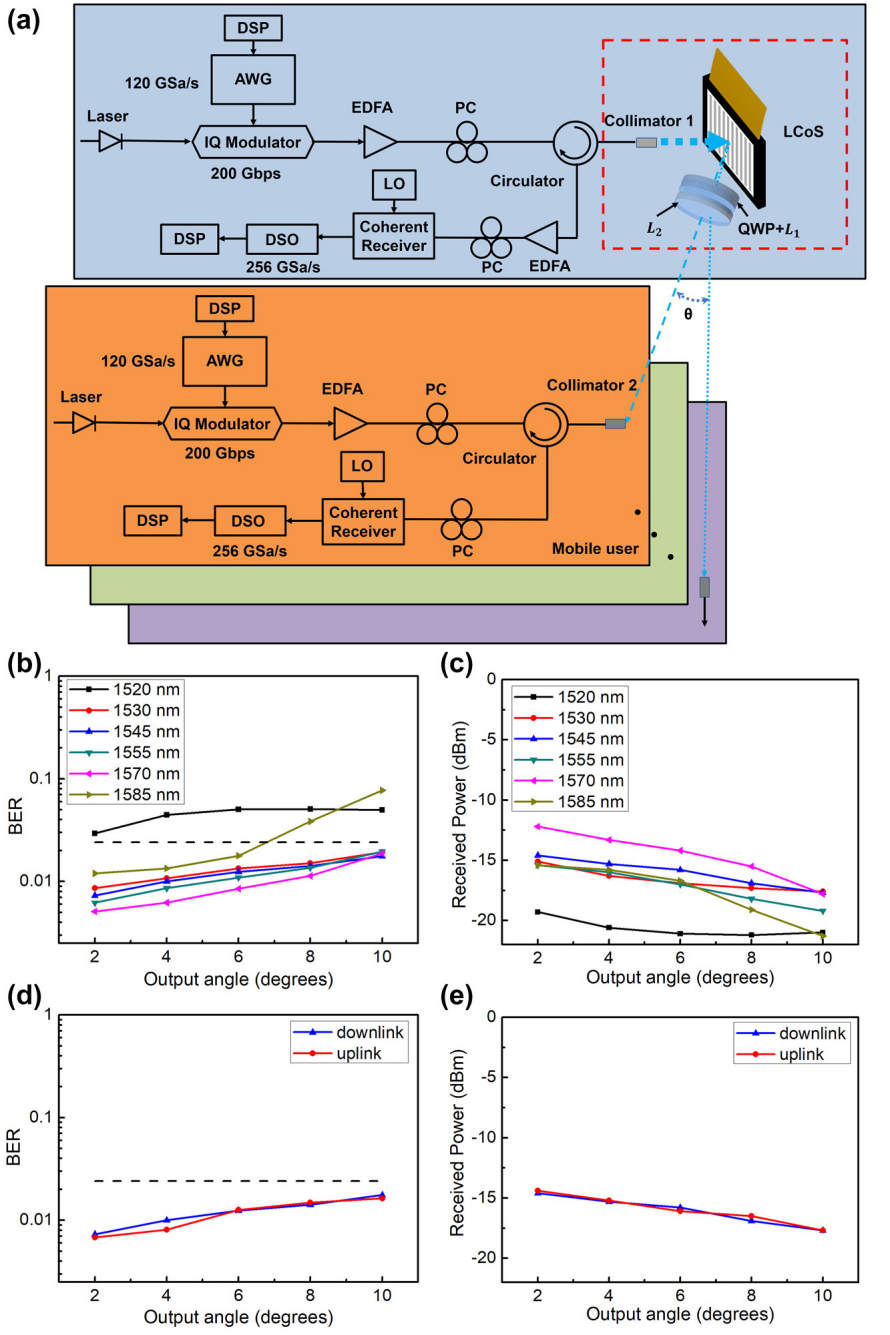
Experimental system configuration and results for the LC metadevice–assisted bidirectional coherent OWC system at the data rate of 200 Gbps for both links. (a) Schematic of the OWC system. (b) Measured BERs and (c) received powers of downlink at different output angles and different wavelengths when the source power is fixed at 12 dBm. (d) Measured BERs and (e) received powers of the downlink and uplink at different output angles and wavelength of 1,545 nm when the source power is fixed at 10 dBm and 2 dB is compensated at the receiving end. The dotted line marks the FEC threshold of 2.4 × 10^−2^.

We applied the fabricated LC metadevice to a bidirectional OWC system for proof-of-concept. Figure 4(a) illustrates the configuration of the experimental system for LC metadevice–assisted bidirectional point-to-point indoor OWC system (more details about the experimental setup of the system are provided in Section S2, Supporting Information).

For the downlink transmission, six wavelengths spanning across S, C, and L band were used as optical carriers to explore full band characteristics of the system. A LiNbO3 in-phase and quadrature-phase (IQ) modulator was driven by an arbitrary waveform generator (AWG) with sampling rate of 120 GSa/s to provide 200 Gbps 16 quadrature amplitude modulation (QAM) signals with a length of 50,000, and each of the six wavelengths carried 200 Gbps signals. After being amplified by an erbium-doped fiber amplifier (EDFA), the optical power outputted from collimator 1 with a beam waist diameter of 1.2 mm was ensured to be ≈12 dBm. To become linear polarization so as to maximize phase modulation efficiency of the polarization-dependent LCoS, the beam was also tuned by a polarization controller (PC) before collimator 1. Thereafter, the beam was illuminated onto the LC metadevice, where the LC metasurface set was positioned closely after the LCoS and aligned its untuned reflected beam axis. As the measured optical loss of LC metadevice was above 2.47 dB, the optical power after the LC metadevice was below 10 dBm, which is under eye-safe operation power. After a transmission distance of 1.5 m in free space, a collimator (collimator 2) with a beam waist diameter of 1.2 mm installed on the multiaxis stage was utilized to couple the optical signal into a single mode fiber. An additional PC was employed to tune the optical signal with single polarization, thus ensuring the single polarization channels of the following digital storage oscilloscope (DSO). The optical signal was converted into electrical signal by the coherent receiver, which was sent to DSO with 256 GSa/s. Finally, the offline digital signal processing (DSP) was executed for BER calculation.

Figure 4(b) shows the downlink BER curves of various 1D unilateral output deflection angles ranging within 10° at different wavelengths of S + C + L band. A wider FOV up to 20° × 20° can be naturally achieved due to the 2D tunability of LCoS and the symmetricity of opposite diffraction orders. For wavelengths of C band (1,530 nm, 1,545 nm, 1,555 nm) and shorter L band (1,570 nm), the LC metadevice presents similar performance for various output deflection angles, and the BER values are below the FEC threshold of 2.4 × 10^−2^ marked with a dotted horizontal line. But for S band (1,520 nm) and longer L band (1,585 nm), the BER values go bad. Figure 4(c) manifests the received power curves of deflection angles and wavelengths corresponding to Figure 4(b), where received powers at the wavelength of 1,530 nm, 1,545 nm, 1,555 nm, and 1,570 nm have similar tendency, while at 1,520 nm and 1,585 nm, the received power go below −20 dBm quickly. This can be contributed to the limited working bandwidth of LCoS device.

For the uplink transmission, the optical wireless system approximates optical reciprocity but has an additional EDFA at the receiving end, as illustrated in Figure 4(a). To ensure the eye safety, the output power of collimator 2 was slightly below 10 dBm. Another EDFA was added at the uplink receiving end to compensate for the 2 dB difference between downlink and uplink transmitting optical powers. As PC can randomly modulate polarization of light in fiber, an LCP beam was transmitted backward from the collimator 2 by tuning the PC at the transmitting end. After passing the LC metasurface set, the beam was transformed into the linear polarized one just aligning with the most modulation efficient polarization of LCoS device. Therefore, the beam was reciprocally transmitted into collimator 1.

Due to the approximately reciprocal optical paths, the optical losses of the uplink transmissions are nearly equal to that of the downlink. Here, we take the user with wavelength of 1,545 nm, for example, using the same modulation format and rate as the downlink ones. Figure 4(e) shows the optical power losses of the uplink and downlink at different output angles at 1,545 nm. It is easy to see that the values of uplink and downlink power losses are close to each other. Take 2° output angle as an example, the total optical power losses are 26.6 dB for downlink and 26.4 dB for uplink. They both include five parts. The first part is caused by the 66.7 % reflection efficiency of LCoS device, which results in a 1.76 dB loss. The second part is the modulation efficiency of LCoS, about 64.3 %, introducing a 1.92 dB loss. The third part is transmission loss of metalens doublet, about a 0.71 dB loss. The fourth part is the coupling loss between the two collimators. For downlink, considering 3 times beam size shrinkage caused by the LC metasurface set, a 1.2 beam waist of collimator 2, a 1.5 m propagation distance, and the wavelength of 1,545 nm, the calculated theoretical downlink coupling loss is 5.26 dB. For uplink, considering 1.1 times beam size expansion caused by the free space propagation, 3 times beam size expansion caused by the LC metasurface set, a 1.2 beam waist of collimator 1, and a 100 mm propagation distance from the LC metasurface set to collimator 1, the theoretical uplink coupling loss is 5.11 dB. The last part is about 16.9 dB (26.4–1.76–1.92–0.71–5.11 = 16.9), which may be attributed to the optical misalignment and the fixed interfacial loss of devices in the optical path. The main loss difference of both links is due to the coupling loss, so the downlink and uplink share nearly the same receiving power. Figure 4(d) shows the BER curves of various deflection angles in the downlink and uplink at the wavelength of 1,545 nm. As can be seen, the uplink BER has similar tendency and values as those of the downlink. The uplink and downlink have approximately the same transmission performance. Therefore, it is feasible to realize a bidirectional high-speed optical wireless communication with tunable large FOV using LC metadevice–assisted system.

## 3 Discussion

The performances of the LC metadevice–assisted OWC system primarily include three parts: FOV, BER, and the operating bandwidth, which can be further improved if the constrains of devices are alleviated. First, the FOV of this system is mainly restricted to the limited steering angle of LCoS device and the aperture and magnification factor of the metalens doublet. The initial FOV may be widened by reducing the pixel size of LCoS, and the broader final FOV is allowed by enlarging LC metasurface apertures. The magnification factor *M* (=−*f*_1_/*f*_2_) can be enlarged by reducing the aperture of lens *f*_2_ and increasing the spacing between metalens doublet. On the one hand, the focal length |*f*_2_| is minimized with allowable minimum effective aperture, as the manufacturable minimum edge period should be no less than 5 μm. On the other hand, an increased spacing distance *d* (=*f*_1_ + *f*_2_) contributes to a longer focal length *f*_1_. However, it should be noted that the effective aperture of lens *f*_2_ is a compromise between larger allowable FOV and larger *M*. Second, the BER performances are closely related to the received powers, and they have the opposite tendency, as clearly exhibited in experimental results shown in Figure 4(b)–(e). Consequently, better BERs need less optical loss. The evitable optical loss is mainly contributed to LCoS devices where bulky package of the metasurface set blocks beam incidence on LCoS at efficient small angles, thus largely limiting the modulation efficiency of LCoS. Therefore, by reducing the package size, the incident angle of light beam onto the LCoS can be smaller; therefore, the modulation efficiency will be improved, and the loss will be greatly suppressed. Third, restricted by the C band operating bandwidth of LCoS device, the communication system has poor performance in S and L band. If an LCoS with broader operating bandwidth is used, the transmission efficiency and BER in S and L band will be enhanced. According to the methods above, the insertion loss caused by devices can be largely reduced, so the distance of free space transmission can have an extra increase without compromising transmission performance.

In addition, the proposed system has potentials to expand to not only the near IR wavelength range, LCP illumination, and small size area. Broader spectrum of wavelengths can be covered by utilizing multiple materials [39], [40]. Additional properties and functionalities are available if meticulously designing the chirality degree of freedom of metasurfaces [41], [42]. For instance, while fixing the spacing distance of the metalenses *d*, magnification factor *M* (=1/(1 − *d*/*f*_1_)) may be altered by adjusting the polarization state of incidence, provided that varying focal lengths of metalenses are engineered through chirality modulation. Furthermore, the LC element fabrication technology used for LC metasurface fabrication has already reached commercialization, paving the way for mass manufacturing. The emergence of other cost-effective and large-scale micro-nanoscale fabrication technologies, such as nanoimprint lithography, transfer printing, and self-assembly–based fabrication [43], [44], [45], can facilitate application of metasurfaces of multiple materials across a wide range of practical uses. Moreover, the LC metadevice–assisted OWC system can not only steer signal beam with magnified angle to users one by one but also broadcast signals to multiple users simultaneously with a larger FOV.

## 4 Conclusions

In this work, we proposed a bidirectional high-speed OWC link with the tunable large FOV assisted by an LC metadevice system. Our method utilizes the intelligent tunability of the LCoS and the angle magnification of metalens doublet. Beam-steering FOV of 20° × 20° is achieved in the OWC system over S/C/L band with data rate of 200 Gbps. To the best of our knowledge, it is the first time to demonstrate a metasurface-based active beam-steering OWC system with tunable increasing steering angle. In addition, the LC metasurfaces are fabricated using an LC element fabrication technology, which has been already commercialized, enabling mass manufacturing. This proposed communication scheme is not necessarily limited to the near IR wavelength range in this work but also can be extended to other wavelength range, such as visible band for underwater OWC and terahertz range for free space communication, which are interconnection scenarios in 6G communication. Compacting the proposed meta-device to chip-scale dimension would open exciting ways for LiDAR for autonomous vehicles and digital holographic display for augmented reality.

## 5 Experimental section

### 5.1 LC metasurface fabrication

The process for fabricating the three LC metasurfaces can be mainly divided into two parts: the preparation of the oriented film and the photopatterning of the designed orientation profiles. Initially, silicon substrates were cleaned with deionized water and isopropyl alcohol, later ultraviolet ozone bathed, and then spin-coated with azo-dye. After heat-cured for several minutes, the azo oriented films were attached firmly, which could be oriented under polarized blue light and guided the orientation of LC directors through intermolecular anchoring energy. Secondly, to realize the designed orientations, a maskless direct laser writing lithography system was introduced, mainly composed of a light source, a polarization controlling module, a reflector, and an optical focusing component. The designed orientation profiles were photopatterned on the oriented film, and later LCs were spin-coated on the oriented film and ultraviolet irradiated. Then the LCP-oriented films with designed orientations were formed. As the films were fragile, substrates with antireflection film were attached on the top to protect them from external impact. For ease of optical operation, the LC QWP and *L*_1_ share the middle substrate to integrate together.

## Supplementary Material

Supplementary Material Details

## References

[1] Khalighi M. A., Uysal M. (2014). Survey on free space optical communication: a communication theory perspective. *IEEE Commun. Surv. Tutor.*.

[2] Uysal M., Nouri H. (2014). Optical wireless communications — an emerging technology. *2014 16th International Conference on Transparent Optical Networks (ICTON)*.

[3] Koonen T. (2018). Indoor optical wireless systems: technology, trends, and applications. *J. Lightwave Technol.*.

[4] Chowdhury M. Z., Hossan M. T., Islam A., Jang Y. M. (2018). A comparative survey of optical wireless technologies: architectures and applications. *IEEE Access*.

[5] Brandl P., Schidl S., Polzer A., Gaberl W., Zimmermann H. (2013). Optical wireless communication with adaptive focus and MEMS-based beam steering. *IEEE Photonics Technol. Lett.*.

[6] Glushko B., Shar A., Medina M., Kin D., Krylov S. (2016). MEMS-based tracking for an indoor optical wireless communication bidirectional link. *IEEE Photonics Technol. Lett.*.

[7] Wang K., Nirmalathas A., Lim C., Alameh K., Skafidas E. (2016). Full-duplex gigabit indoor optical wireless communication system with CAP modulation. *IEEE Photonics Technol. Lett.*.

[8] Feng F., White I. H., Wilkinson T. D. (2013). Free space communications with beam steering a two-electrode tapered laser diode using liquid-crystal SLM. *J. Lightwave Technol.*.

[9] You Q., Luo M., Xiao X., Yu S. (2020). 2D optical wireless broadcasting system enabled by a liquid crystal on silicon and rotated-splitting-SLM algorithm. *Opt. Express*.

[10] Wang G. (2018). Highly efficient optical beam steering using an in-fiber diffraction grating for full duplex indoor optical wireless communication. *J. Lightwave Technol.*.

[11] Oh C. W., Tangdiongga E., Koonen A. M. J. (2014). Steerable pencil beams for multi-Gbps indoor optical wireless communication. *Opt. Lett.*.

[12] Koonen T., Oh J., Mekonnen K., Cao Z., Tangdiongga E. (2016). Ultra-high capacity indoor optical wireless communication using 2D-steered pencil beams. *J. Lightwave Technol.*.

[13] Yu N. (2011). Light propagation with phase discontinuities: generalized laws of reflection and refraction. *Science*.

[14] Tao J. (2021). Light spin angular momentum spatial mode converter based on dielectric metasurface. *J. Lightwave Technol.*.

[15] Wu L., Tao J., Zheng G. (2018). Controlling phase of arbitrary polarizations using both the geometric phase and the propagation phase. *Phys. Rev. B*.

[16] Chen R., Chang Y.-K., Zhuang Z.-p., Liu Y., Chen W.-J., Dong J.-w. (2023). Metasurface-based fiber-to-chip multiplexing coupler. *Adv. Opt. Mater.*.

[17] Dong Y., Wang Z., Xiong C., Deng B., Hu B. (2023). Printable and low-cost perfect terahertz absorber realized by a laser-induced graphene metasurface. *Opt. Lett.*.

[18] Lee D. (2024). Wide field-of-hearing metalens for aberration-free sound capture. *Nat. Commun.*.

[19] Kim J. (2024). Dynamic hyperspectral holography enabled by inverse-designed metasurfaces with oblique helicoidal cholesterics. *Adv. Mater.*.

[20] Jeon D., Rho J. (2024). Quasi-trapped guided mode in a metasurface waveguide for independent control of multiple nonlocal modes. *ACS Photonics*.

[21] Deng L. (2023). Full complex-amplitude engineering by orientation-assisted bilayer metasurfaces. *Adv. Opt. Mater.*.

[22] Zhang J. C. (2024). Programmable optical meta-holograms. *Nanophotonics*.

[23] Kang D. (2024). Liquid crystal-integrated metasurfaces for an active photonic platform. *Opto-Electron. Adv.*.

[24] Yang Y. (2023). Integrated metasurfaces for re-envisioning a near-future disruptive optical platfrom. *Light: Sci. Appl.*.

[25] Moon S. Tutorial on metalenses for advanced flat optics: design, fabrication, and critical considerations. *J. Appl. Phys.*.

[26] Kim J., Seong J., Yang Y., Moon S., Badloe T., Rho J. (2022). Tunable metasurfaces towards versatile metalenses and metaholograms: a review. *Adv. Photonics*.

[27] Kim J., Yang Y., Badloe T., Kim I., Yoon G., Pho J. (2021). Geometric and physical configurations of meta-atoms for advanced metasurface holography. *InfoMat*.

[28] Nazemosadat E. (2019). Dielectric broadband metasurfaces for fiber mode-multiplexed communications. *Adv. Opt. Mater.*.

[29] Kruk S. (2018). Transparent dielectric metasurfaces for spatial mode multiplexing. *Laser Photonics Rev.*.

[30] Huang J. (2021). A 20-gbps beam-steered infrared wireless link enabled by a passively field-programmable metasurface. *Laser Photonics Rev.*.

[31] Tao J. (2022). Mass-manufactured beam-steering metasurfaces for high-speed full-duplex optical wireless-broadcasting communications. *Adv. Mater.*.

[32] Tao J. (2023). Beam-steering metasurfaces assisted coherent optical wireless multichannel communication system. *Nanophotonics*.

[33] Hu J. (2024). A metasurface-based full-color circular auto-focusing Airy beam transmitter for stable high-speed underwater wireless optical communications. *Nat. Commun.*.

[34] Tao J. (2023). Beam-steering metadevices for intelligent optical wireless-broadcasting communications. *Adv. Photonics Res.*.

[35] Li S., Xu X., Maruthiyodan Veetil R., Valuckas V., Paniagua‐Domínguez R., Kuznetsov A. I. (2019). Phase-only transmissive spatial light modulator based on tunable dielectric metasurface. *Science*.

[36] Yang Z., Liu M., Komar A., Xu L., Neshev D. N. (2022). Phase-only tuning of extreme huygens metasurfaces enabled by optical anisotropy. *Adv. Opt. Mater.*.

[37] Zhang L. (2023). Highly tunable cascaded metasurfaces for continuous two-dimensional beam steering. *Adv. Sci.*.

[38] Zhang J. C. (2023). A 6G meta-device for 3D varifocal. *Sci. Adv.*.

[39] Yang Y. (2023). Revisiting optica material platforms for efficient linear and nonlinear dielectric metasurfaces in the ultraviolet, visible, and infrared. *ACS Photonics*.

[40] Yang Y. Revealing structural disorder in hydrogenated amorphous silicon for a low-loss photonic platform at visible frequencies. *Adv. Mater.*.

[41] Mun J. (2020). Electromagnetic chirality: from fundamentals to nontraditional chiroptical phenomena. *Light: Sci. Appl.*.

[42] Yang Y., Kim H., Badloe T., Rho J. (2022). Gap-plasmon-driven spin angular momentum selection of chiral metasurfaces for intensity-tunable metaholography working at visible frequencies. *Nonophotonics*.

[43] Seong J., Jeon Y., Yang Y., Badloe T., Rho J. (2024). Cost-effective and environmentally friendly mass manufacturing of optical metasurfacs towards practical applications and commercialization. *Int. J. Precis. Manuf. -Green Technol.*.

[44] Kim Y., Kim H., Yang Y., Badloe T., Jeon N., Rho J. (2022). Three-dimensional artificial chirality towards low-cost and ultra-sensitive enantioselective sensing. *Nanoscale*.

[45] Kang H. (2023). Emerging low-cost, large-scale photonic platform with soft lithography and self-assembly. *Photonics Insights*.

