# Supplementary material for: Bidirectional high-speed optical wireless communication with tunable large field of view assisted by liquid crystal metadevice
Source: Nanophotonics. 2024 Sep 27;13(23):4347–56. doi: 10.1515/nanoph-2024-0434 (PMC11635923; doi:10.1515/nanoph-2024-0434)
Supplement: Supplementary file 1 — Supplementary Material Details [file j_nanoph-2024-0434_suppl_001.docx]

Supporting Information

Bidirectional high-capacity optical wireless communication with tunable large field of view assisted by liquid crystal metadevice

Mian Wu^1^, Chao Yang^1,2^, Yuhan Gong^1^, Lin Wu^1^, Ming Luo^1,2^, Ying Qiu^1^, Yongquan Zeng^2,3^, Zile Li^2,3^, Zichen Liu^2^, Chao Li^2^, Hanbing Li^1^, Xi Xiao^2,4^, Zhixue He^2^, Guoxing Zheng^2,3^, Shaohua Yu^2*^ and Jin Tao^1,2*^

^1^State Key Laboratory of Optical Communication Technologies and Networks, China Information Communication Technologies Group Corporation (CICT), Wuhan 430074, China.

^2^Peng Cheng Laboratory, Shenzhen 518055, China

^3^Electronic Information School, Wuhan University, Wuhan 430072, China.

^4^National Information Optoelectronics Innovation Center, Wuhan 430074, China

*e-mail: yush@cae.cn, [taojin@cict.com](mailto:taojin@cict.com)

**S1. Videos for beam spot moving process**

**Video 1.** The beam spot moving process modulated by LCoS only.

**Video 2.** The beam spot moving process modulated by the whole LC metadevice.

In order to observe the movement process, we set a time delay in the moving. The actual response time is in ms.

**S2. The experimental configuration of the LC metadevice-assisted bidirectional point-to-point indoor OWC system**

The experimental setup for the OWC system in this work is depicted in **Figure S1**a. The system is primarily divided into two parts: the signal generation and processing part, and the free-space optical communication part. For signal generation, conventional DSP, tunable lasers, an IQ modulator, an AWG, and an EDFA are employed. The generated signal is then transmitted through a PC located on the right optical stage, as shown in Figure S1a, and subsequently emitted by fiber collimator 1. Figure S1b provides a close-up view of the free space optical part. Here, the optical beam is modulated by an LC metadevice, traverses a 1.5 m distance in free space, and is ultimately captured by collimator 2, which is mounted on a multi-axis stage. Further details of receiving end and transmitting end of the free space optical part are illustrated in Figure S1c and S1d respectively. As illustrated in Figure S1d, the LCoS is positioned at a 45^°^ angle to both the orientation of collimator 1 and the optical axis of the LC metasurface set. Once the signal is captured by collimator 2, the coherent receiver converts the optical signal back into an electrical signal. This signal is then relayed to a DSO and finally processed by the offline DSP.


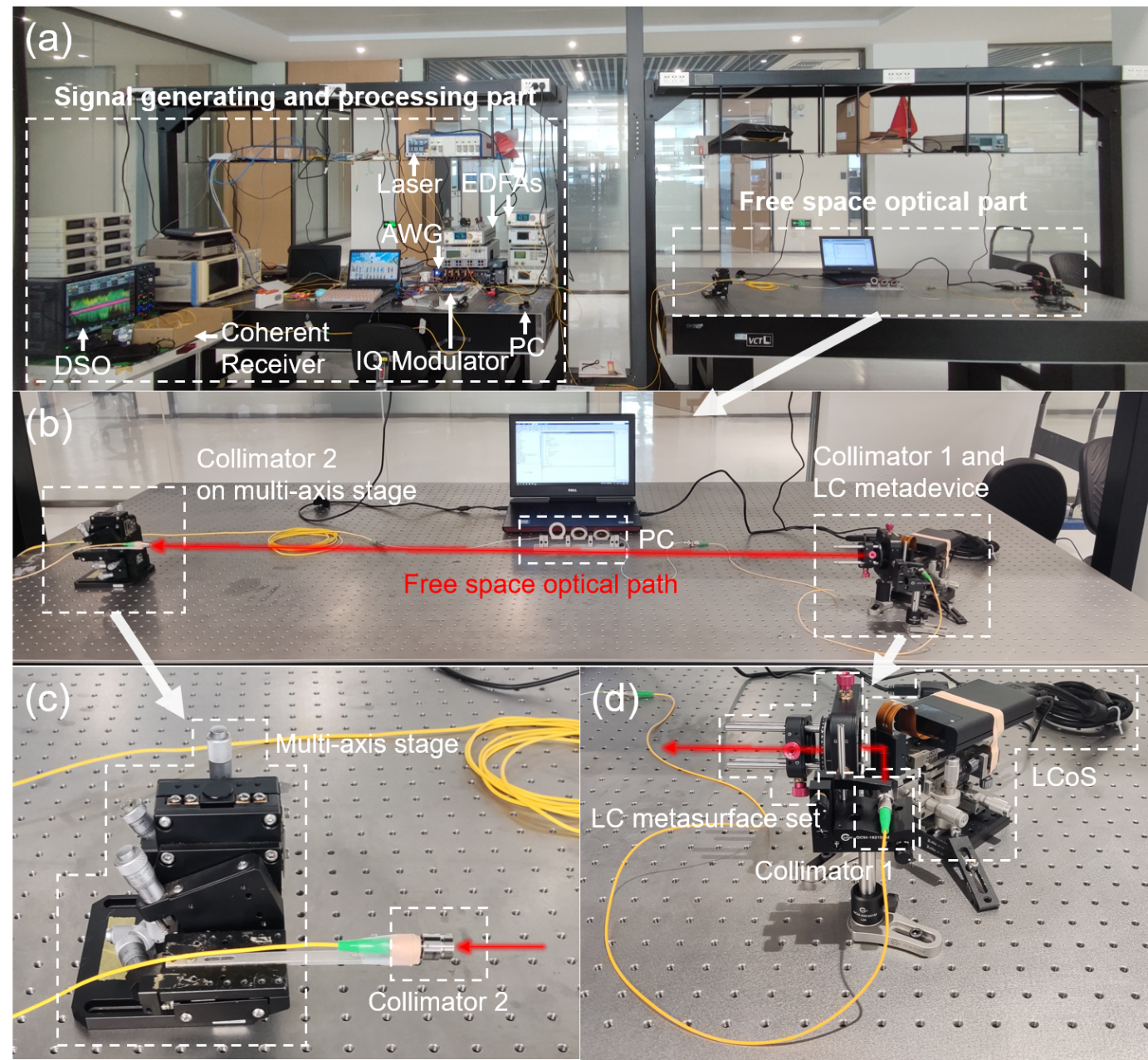


**Figure S1.** Experimental configuration for the LC metadevice assisted bidirectional indoor OWC system. (a) The overall experimental configuration. (b) The enlarged picture of free space optical part. (c) Details of collimator 2 on multi-axis stage to receive the downlink signal beam. (d) Details of collimator 1 and LC metadevice to modulate and transmit the downlink signal beam.
